# High-energy diffuse X-ray scattering at ultra-small-angle grazing incidence for local structure study of single-crystalline thin films

**DOI:** 10.1107/S1600576725005837

**Published:** 2025-07-29

**Authors:** Joohee Bang, Nives Strkalj, Martin F. Sarott, Yevheniia Kholina, Morgan Trassin, Thomas Weber

**Affiliations:** a ETH Zürich, Laboratory of Multifunctional Ferroic Materials, Vladimir-Prelog-Weg 1-5/10, 8093 Zürich, Switzerland; b Center for Advanced Laser Techniques, Institute of Physics, Bijenička Cesta 46, 10000 Zagreb, Croatia; c Zernike Institute for Advanced Materials, University of Groningen, Nijenborgh 3, 9747AG Groningen, The Netherlands; d Groningen Cognitive Systems and Materials Center (CogniGron), University of Groningen, Nijenborgh 3, 9747AG Groningen, The Netherlands; e ETH Zürich, Department of Materials, X-ray Platform, Vladimir-Prelog-Weg 1-5/10 8093 Zürich, Switzerland; Institut de Recherche sur les Céramiques, France

**Keywords:** diffuse scattering, synchrotron X-rays, high-energy studies, single crystals, thin films, grazing incidence

## Abstract

We present a novel experimental approach employing high-energy X-ray scattering in ultra-small-angle grazing-incidence geometry to investigate local atomic structures in single-crystalline thin films. This non-destructive and non-invasive method overcomes the limitations of conventional moderate-energy grazing-incidence diffraction, achieving both high reciprocal-space resolution and coverage and high surface sensitivity. By leveraging high-energy X-ray diffraction, we enable quantitative analysis of local structures in the model system of ferroelectric PbTiO_3_ and dielectric SrTiO_3_ superlattices through three-dimensional difference pair distribution function analysis. The approach provides detailed insights into atomic structures in single-crystalline thin films with local order, capturing information on spatial correlations within and across unit cells.

## Abstract 2

High-energy X-ray scattering in an ultra-small-angle grazing-incidence geometry is employed for local structure analysis of epitaxially grown single-crystalline thin films with atomic scale resolution. This novel approach allows for non-invasive, non-destructive and quantitative 3D analysis of local order in thin films, providing a valuable complement to traditional solid-state characterization techniques.

## 1. Introduction

Single-crystalline oxide thin films exhibit rich diversity in physical properties, making them highly attractive for the development of energy-efficient electronic devices. The ongoing demand for miniaturization in microelectronics has spurred extensive research into tailoring properties in the ultra-thin regime (Schlom *et al.*, 2014 ▸; Li *et al.*, 2018 ▸; Strkalj *et al.*, 2019 ▸). This drive underscores the necessity for a comprehensive understanding of structure from the macroscale to atomic resolution and its correlation with the physical behavior of the films.

Ferroelectric oxide thin films, for instance, display electrically switchable polarization, often accompanied by high dielectric constants (Quan Jiang *et al.*, 2015 ▸; Pandya *et al.*, 2017 ▸), and their switching properties are greatly influenced by the existence of polarization domains and their reorientation (Setter *et al.*, 2006 ▸; Tagantsev *et al.*, 2010 ▸; Martin & Rappe, 2016 ▸; Evans *et al.*, 2020 ▸). Recently, complex orderings such as polar vortex–antivortex pairs and polar skyrmions (Yadav *et al.*, 2016 ▸; Das *et al.*, 2019 ▸) were observed in engineered superlattices of ferroelectric PbTiO_3_ and dielectric SrTiO_3_. The discovery of such exotic phases called for understanding the domain architecture with atomic resolution, as chirality determines their properties such as stability and direction of motion under an external field (Zubko, 2019 ▸).

Structural characterization of the superlattices has been primarily conducted with imaging techniques such as scanning transmission electron microscopy (Petford-Long & Chiaramonti, 2008 ▸) and scanning probe microscopy (Bian *et al.*, 2021 ▸). Although microscopic methods offer high-resolution maps of atomic structures and are adept at probing nanoscale domains, they are often invasive, necessitating destructive sample preparation. The data obtained from these techniques are typically confined to very small regions of the sample and cross sections with limited depth information, which may not accurately represent the structural and physical properties of the macroscopic sample. Therefore, the limited three-dimensional resolution of these methods hinders a holistic understanding of domain architectures.

On the one hand, non-invasive and non-destructive investigations of thin films are commonly conducted with X-ray-diffraction-based methods such as X-ray reflectivity, reciprocal-space mapping or symmetric θ–2θ scans (Yadav *et al.*, 2016 ▸; Strkalj *et al.*, 2021 ▸; Tan *et al.*, 2021 ▸). These methods are routinely used as sample averaging tools for investigating the thicknesses, roughness and electron density, as well as the out-of-plane and in-plane lattice parameters of a given film. Furthermore, they provide microstructural insights including epitaxial matching, domain sizes, mosaicity *etc.* However, they cover limited volumes of reciprocal space and are therefore not able to reveal local order information with atomic resolution. Recently, larger reciprocal-space volumes of superlattices have been measured with synchrotron radiation (Zatterin *et al.*, 2024 ▸). Nevertheless, the limited reciprocal-space coverage and resolution of the reported experiment still restricts a comprehensive investigation of the local atomic order.

The use of large-volume diffuse X-ray scattering has proven to be a powerful tool for the investigation of local atomic order in *bulk* single crystals, especially for quantitative characterization of local order properties (Welberry & Weber, 2016 ▸). The method is non-invasive and non-destructive, and provides a three-dimensional view of sample-averaged local order properties with atomic resolution. Instead of directly mapping individual atoms, it yields statistical insights into atomic pair correlation parameters, which makes it perfectly complementary to the aforementioned microscopy- and X-ray-based methods.

While the method holds great promise for studying local atomic structure in thin films, to the best of our knowledge, a corresponding analysis with high reciprocal-space resolution and large coverage has yet to be conducted. Collecting three-dimensional diffuse scattering data from thin films presents a number of unique challenges as the experimental strategies used for bulk single crystals can not be directly transferred. This is largely because thin films are typically grown on substrates that are orders of magnitude thicker than the film itself, posing a significant challenge in separating the film and bulk signals. In addition, the highly anisotropic shape of the sample leads to severe limitations in orienting the crystals relative to the beam and strong shadowing effects, which requires making compromises in reciprocal-space resolution.

In this work, we investigate the potential of three-dimensional diffuse X-ray scattering analysis for examining local structures in thin films, with a focus on the comparison of moderate- and high-energy grazing-incidence X-ray experiments. The comparison is motivated by the capabilities of high-energy grazing-incidence measurements in extending reciprocal-space coverage and thus improving real-space resolution. In fact, the advantages of high-energy X-ray diffraction in the structural investigation of thin-film structures have been previously recognized, especially in studies of polycrystalline thin films (Jensen *et al.*, 2015 ▸; Nakamura *et al.*, 2017 ▸; Shi *et al.*, 2017 ▸; Dippel *et al.*, 2019 ▸). This paper demonstrates an experimental approach for the collection and processing of three-dimensional diffuse scattering data for quantitative studies of local structures in single-crystalline thin films as a proof of principle. We employ high-energy synchrotron X-rays in an ultra-small-angle grazing-incidence geometry to collect three-dimensional diffuse scattering data for superlattices of ferroelectric PbTiO_3_ (PTO) and dielectric SrTiO_3_ (STO) as model systems.

## 2. Experimental

### 2.1. Sample preparation

A superlattice composed of epitaxially grown, alternating layers of a five-unit-cell-thick ferroelectric PTO layer and a five-unit-cell-thick dielectric STO layer, repeated for a total of 12 bilayers, was grown on a single-crystalline (001)-oriented STO substrate (5 × 5 mm, Crystec GmbH) using pulsed-laser deposition (PLD). The in-plane lattice constants of ferroelectric PTO and dielectric STO are *a* = 3.904 Å (Nomura & Mitsui, 1981 ▸) and *a* = 3.905 Å (Mabud & Glazer, 1979 ▸), respectively, and hence the two layers are well lattice matched in plane. Prior to superlattice growth, a ten-unit-cell-thick conducting SrRuO_3_ (SRO) layer was deposited to serve as a buffer and bottom electrode for subsequent piezoresponse force microscopy measurements, which are not discussed in this work. The superlattice deposition began with a PTO layer and ended with an extra PTO capping layer on top of the 12-bilayer stack. A high-power pulsed KrF excimer laser (wavelength 248 nm, pulse duration 20 ns) was used for the deposition, with reflection high-energy electron diffraction employed *in situ* to monitor the two-dimensional growth with unit-cell precision.

For the growth of the layers, the STO substrate was kept at 700°C for SRO deposition and 550°C for PTO and STO deposition. The oxygen partial pressure was 0.12 mbar for all layers. The laser fluence was set to 0.7 J cm^−2^ for SRO growth and 0.8 J cm^−2^ for PTO and STO growth, with repetition rates of 2 Hz for SRO, 4 Hz for PTO and 2 Hz for STO.

### 2.2. Data acquisition and treatment

**Figure 1:**
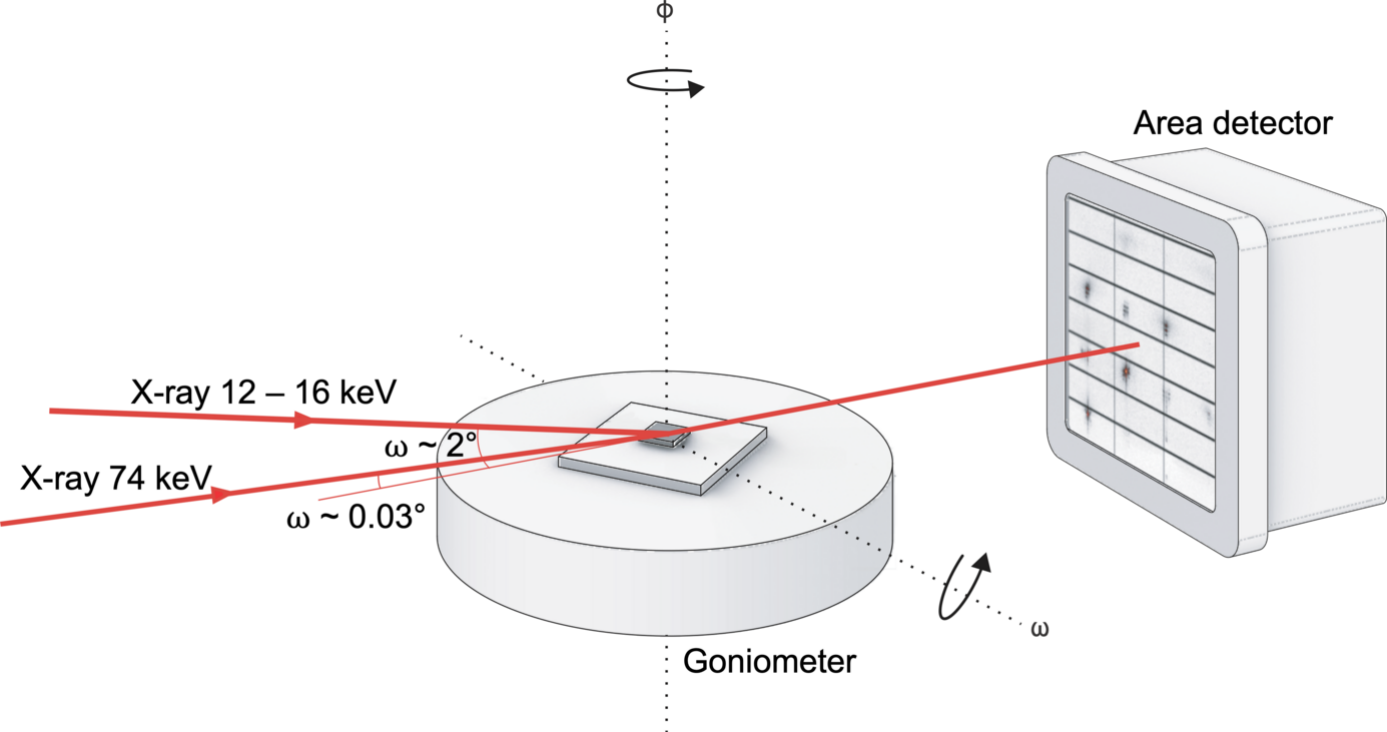
Schematic diagram of the ME-GID setup on the BM01 beamline at ESRF and the HE-GID setup on the P07 beamline at PETRA III, DESY. The ϕ axis is horizontal on BM01 and vertical on P07.

In order to collect three-dimensional diffuse scattering data of the film, a conventional moderate-energy grazing-incidence X-ray diffraction (ME-GID) experiment was first conducted on beamline BM01 at ESRF, Grenoble, France. The data were collected with X-ray energies of 12.8 keV (λ = 0.969 Å, absorption coefficients μ_STO,12.8keV_ = 148 cm^−1^, μ_PTO,12.8keV_ = 421 cm^−1^; all absorption coefficients are approximated by their bulk properties and were calculated using https://11bm.xray.aps.anl.gov/absorb/, while absorption edges were taken from https://xraydb.xrayabsorption.org/) and 15.9 keV (λ = 0.780 Å, μ_STO,15.9keV_ = 80 cm^−1^, μ_PTO,15.9keV_ = 804 cm^−1^) on a PILATUS 2M pixel area detector with a pixel size of 172 µm and a 1475 (horizontal) × 1679 (vertical) pixel array. The ME-GID experiment with lower energy was measured below the *K* and *L* absorption edges of Sr (*K* edge 16.1 keV) and Pb (*L*_3_ edge ∼13.0 keV, *L*_1_ edge 15.86 keV), respectively, while the experiment with higher energy was above and in particular close to the *L*_1_ edge of Pb. The size of the beam at the sample was 0.5 × 0.5 mm and the beam divergence was 0.2°. The rotational step-scan measurements were conducted on a κ-diffractometer at sample-to-detector distances (SDDs) of 143 and 241 mm and at 130 K to minimize thermal diffuse scattering (TDS) from the substrate. The measurements were acquired in two different geometries with rotation axes around ϕ and ω, respectively, to collect a comprehensive reciprocal-space volume (Fig. 1 ▸). For ϕ scans, the sample was rotated 360° around its surface normal with the ϕ axis in a horizontal orientation and a step size per frame of 0.1°. The incident angle of the beam relative to the surface of the sample was 2°. The sample was aligned with the surface parallel to the direct beam using standard techniques, *i.e.* by rotating the sample along its two main axes with their respective orientation perpendicular to the beam until the transmission was at its highest. In the ϕ scans, only about half of the detector area could be utilized in the direction of the axis of rotation due to geometric limitations, while the other half was shadowed by the sample. For ω scans, in which the incident tilt angle of the primary beam was varied, the step size was 0.1° with a scan range of 30° starting from ω = 0°, in which the sample was parallel to the beam. In all measurements, the exposure time was 1 s per frame.

High-energy grazing-incidence X-ray diffraction (HE-GID) experiments were conducted on beamline P07 at PETRA III, DESY, Hamburg, Germany. The data were collected with an X-ray energy of 74 keV (λ = 0.168 Å, μ_STO,74keV_ = 4.24 cm^−1^, μ_PTO,74keV_ = 13.7 cm^−1^) using a PILATUS3 X CdTe 2M pixel area detector with a pixel size of 172 µm and a 1475 (horizontal) × 1679 (vertical) pixel array. The beam was focused on the sample with concave X-ray lenses leading to a convergence angle of ∼0.003°. The lateral and vertical lengths of the beam at the sample position were 30 and 2 µm, respectively. The sample alignment was done by analogy with the procedure for the ME-GID experiment. The alignment scan profiles indicated high parallelism with an accuracy better than 0.004°, which is essentially limited by the primary beam convergence angle. Once the parallel orientation of the sample relative to the beam was identified, the optimal height of the sample was determined by gradually shifting the sample across the beam until a halving of the primary beam intensity was observed. Data were collected at room temperature at SDDs of 493.7 mm for large reciprocal-space coverage and 850 mm for high resolution. The sample was rotated 200° around its surface normal with the ϕ axis in a vertical orientation and an incident angle of 0.03°, which is about an order of magnitude larger than the beam convergence angle of 0.003°. In contrast to the ME-GID measurements, data collection scans were repeated with the detectors shifted by the width of the gap between the detector modules to fill in the missing information. Parameters for both ME-GID and HE-GID experiments are compared in Table 1 ▸.

The determination of an accurate orientation matrix, which is required for reciprocal-space reconstruction, is challenging for epitaxially grown thin films. Due to the similarity of the substrate and film lattices, their signals are difficult to separate. Furthermore, the intensity of the substrate reflections is significantly stronger than that of the thin films, resulting in strong saturation of most substrate peaks under normal diffuse scattering measurement conditions. This renders them unsuitable for the precise determination of the orientation matrix. To measure unsaturated substrate reflections and almost completely suppress the film signals, we conducted additional HE-GID measurements with strongly attenuated beams. This approach facilitated the acquisition of high-quality orientation matrices with the *X-ray Detector Software* (*XDS*) program (Kabsch, 2010 ▸), which could then be used to calculate three-dimensional reciprocal-space reconstructions with the *Meerkat* program package (Simonov, 2015 ▸). As the orientation matrix was determined on the basis of the substrate, all subsequent reciprocal-space coordinates refer to the substrate STO structure (cubic, *a* = 3.905 Å).

## 3. Results and discussion

### 3.1. Structure-related observations

**Figure 2:**
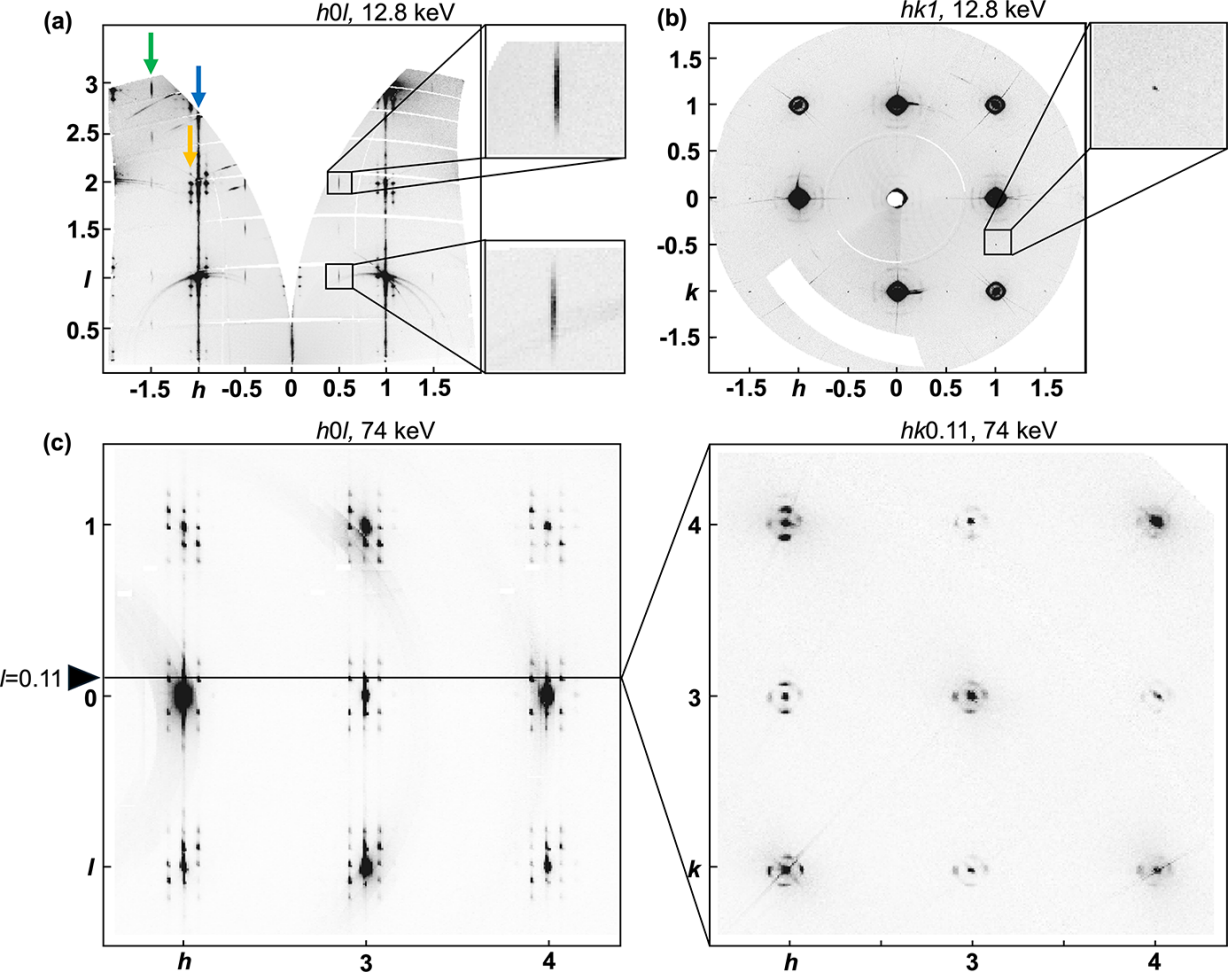
Reconstruction of (*a*) *h*0*l* and (*b*) *hk*1 layers, which were collected with an X-ray energy of 12.8 keV at an incident angle of 2° and an SDD of 241 mm. In panel (*a*), the crystal truncation rods (CTRs) are visible at each integer *h*, marked by the blue arrow. The insets show the sharp streaks oriented along c*, observed at every half-integer *h* as indicated by the green arrow. Additionally, satellite peaks near the CTRs are noted with the yellow arrow. In panel (*b*), diffuse rings are shown around the CTRs and the inset shows the cross section of the half-integer streaks from panel (*a*) with adjusted gray contrast to enhance visual clarity. (*c*) Reconstruction of the *h*0*l* reciprocal-space layer on the left, where the satellite peaks around the CTRs in every integer *k* are cross sections of the strongly modulated diffuse rings shown in the *hk*0.11 layer on the right. Both reciprocal-space layers were collected at an SDD of 850 mm with an X-ray energy of 74 keV.

Reconstructions of total scattering data collected from both series of measurements show three structure-related features: crystal truncation rods (CTRs), which are the Bragg reflections from the thin films [Fig. 2 ▸(*a*), blue arrow], along with diffuse scattering from the local atomic structure, as well as Bragg peaks and TDS from the substrate. This last can be subdivided into two sets of features. Strongly intensity-modulated diffuse rings around the CTRs represent in-plane domain structures arising from the strained film [Fig. 2 ▸(*a*), yellow arrow]. Periodic patterns of sharp streaks with orientation along c* are present at every half-integer *h*, *k* and full- and half-integer *l* positions [Fig. 2 ▸(*a*), green arrow], which are associated with a unit-cell doubling due to a correlated tilt pattern of the oxygen octahedra (Glazer, 1975 ▸; Woodward & Reaney, 2005 ▸), with long-range order along the in-plane directions and limited correlation length along the out-of-plane direction.

### 3.2. ME-GID

**Figure 3:**
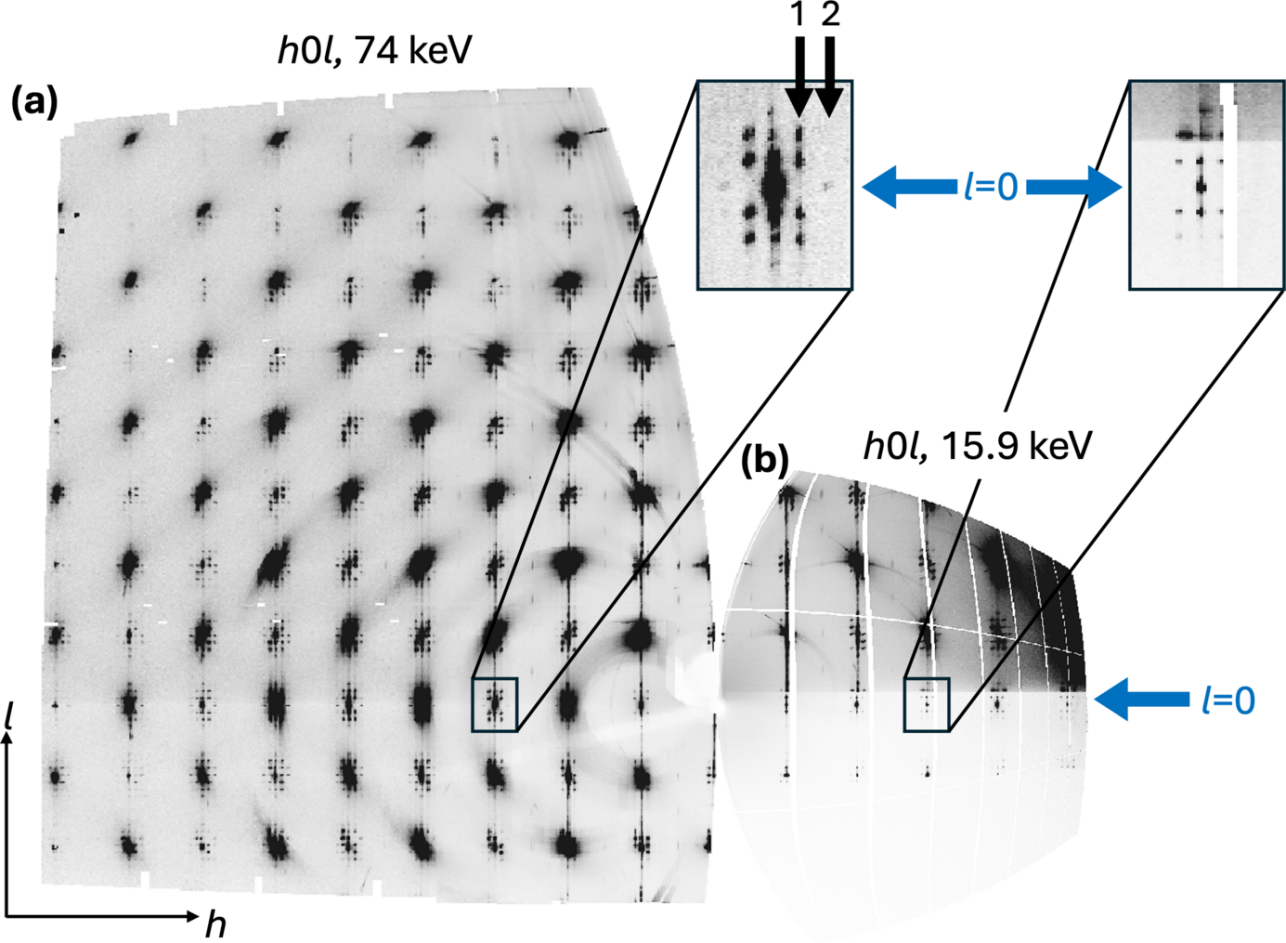
(*a*) Reconstruction of *h*0*l* layers collected at an SDD of 493.7 mm, an incident angle of 0.03° and an X-ray energy of 74 keV, with detector gaps filled (HE-GID). (*b*) Reconstruction of *h*0*l* layers collected at an SDD of 143 mm, an incident angle of 2° and an X-ray energy of 15.9 keV (ME-GID). Here, the detector gaps are not filled. The magnified insets show the corresponding reflection environments from the two experiments. In these insets, the HE-GID recorded 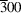 pattern reveals both the absence of the first-order ring shown with arrow 1 and the presence of a weak second-order ring shown with arrow 2, whereas most of the ME-GID 300 environment is heavily shadowed by the substrate, making it impossible to observe such details.

In addition to the intrinsic features arising from the structure of the PTO/STO superlattice, artifacts deriving either from the sample or from the instrument were observed in the scattering data. For one, the data exhibit a marked reduction in signal intensity below and slightly above the *l* = 0 layer, attributed to signal attenuation by the sample. This phenomenon is illustrated in Figs. 3 ▸ and 4 ▸(*a*). The observation horizon at *l* ≃ 0 is directly influenced by the incident angle ω of the beam relative to the sample surface. Specifically, any scattered signal with an out-of-plane diffraction angle 2θ_⊥_ < ω needs to penetrate the substrate and is strongly attenuated. The minimum observable unshaded out-of-plane signal can thus be calculated as 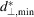
 = 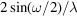. Given ω= 2° and λ = 0.780 Å, 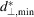 = 0.045 Å^−1^ or 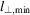 = 0.17 with respect to the reciprocal substrate structure.

**Figure 4:**
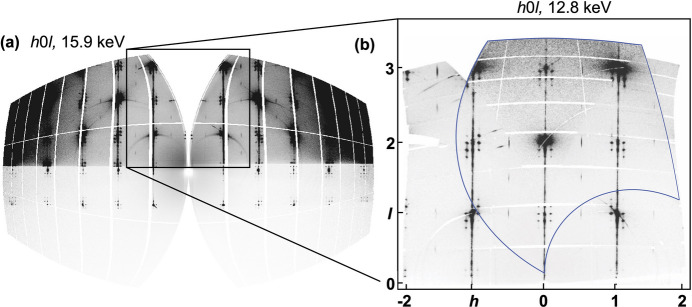
(*a*) Reconstruction of *h*0*l* layers from a ϕ scan collected with an X-ray energy of 15.9 keV at an incident angle of 2° and an SDD of 143 mm. The strong background in the reconstruction especially prominent at high diffraction angles is due to fluorescence scattering. (*b*) Merging of a high-resolution ϕ-scan reconstruction and an ω-scan reconstruction that is absorption corrected and scaled to ϕ. Both scans were collected with 12.8 keV and at an incident angle of 2° and an SDD of 241 mm. The region indicated with a blue outline shows the coverage of the ω-scan reconstruction. Note that the grayscale contrast of the merged reconstruction has been lowered to show the overlap better. Panel (*b*) is not a magnified view of panel (*a*), but a reconstruction from measurements at different wavelengths in the range indicated by the boxed region of panel (*a*). The merging of ϕ and ω scans at 12.8 keV is shown in the inset as the ω scans at 15.9 keV were not measured.

Secondly, a V-shaped blind region is observed on the 00*l* axis [Fig. 4 ▸(*a*)], which is a feature characteristic of single-axis scan measurements. The orientation of the gap is defined by the direction of the rotation axis, while the opening is determined by the curvature of the Ewald sphere. In order to fill the gap, ME-GID experiments were performed in which the rotation axis was positioned within the film layer (ω scans). In contrast to ϕ scans, where the incident angle and consequently the surface sensitivity of the experiment remain constant regardless of the rotation angle, the surface sensitivity variation of ω scans becomes significant as each of the frames is taken at a different incident angle. Since the pathway of the X-rays in the film becomes shorter as the incident angle increases, the recorded signals become weaker and require a corresponding correction. To address this, the ω-scan frames were pixel-wise multiplied with the correction factor 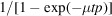 (Birkholz, 2019 ▸), where μ is approximated as the average absorption factor of the layers, *t* = 48 nm is the thickness of the entire film and *p* represents the beam path within the sample and is defined as 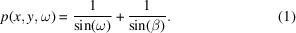
The first term corresponds to the incident beam path and the second term represents the scattered beam path. The angle β is calculated to effectively incorporate the in-plane component of the diffracted beam by defining **k**_diffracted_ as 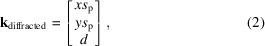
where *x* and *y* denote the pixel position on the area detector for the diffracted beam relative to the incident point of the direct beam, *s*_p_ is the pixel size of the detector, and *d* represents the SDD. The vector **k**_init_ is defined as 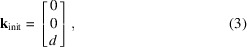
serving as a reference vector aligned with the primary beam to facilitate the calculation of β. We then calculate 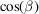 as 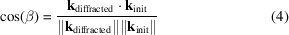
and obtain 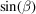 to determine the final diffracted beam path. Following absorption correction, the reconstructions of the ϕ and ω scans were scaled by minimizing the differences in the intensity of the pixels using a linear least-squares method and were subsequently merged [Fig. 4 ▸(*b*)].

**Figure 5:**
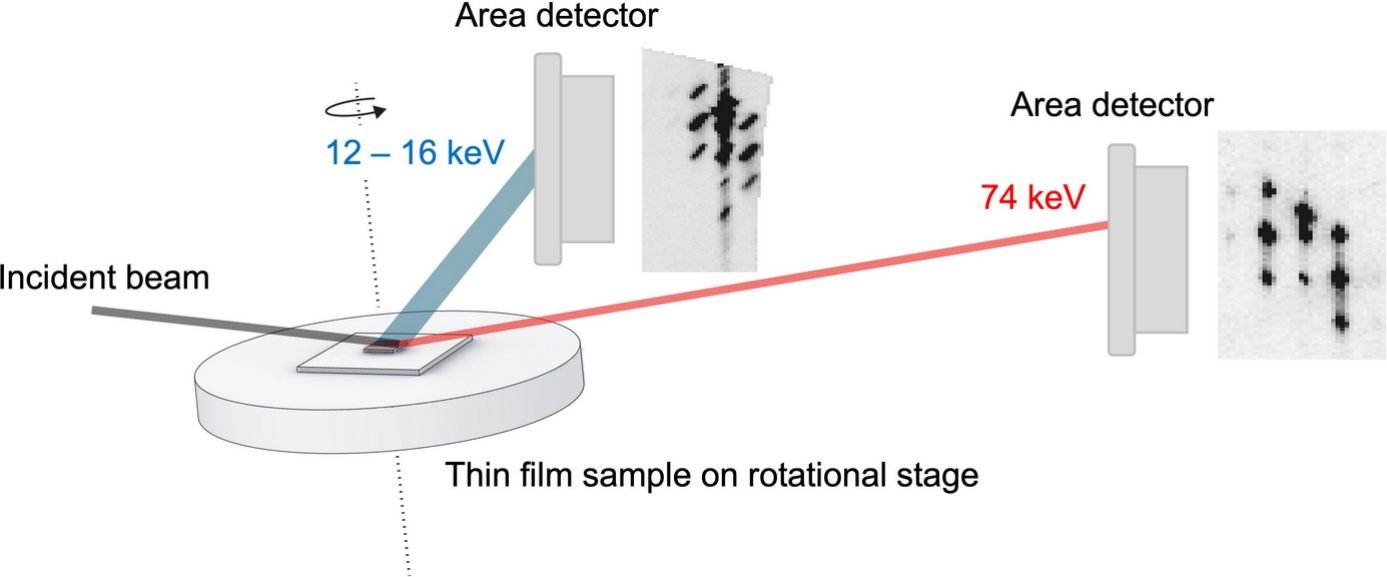
Schematic comparison of ME-GID (blue) and HE-GID (red) experiments with the same incident angle. The large scattering angle of the moderate-energy X-rays leads to a large footprint of the beam on the detector, causing a smearing effect as shown for the example of the 032 reflection environment. The small scattering angle of the high-energy X-rays with sufficiently long SDD significantly improves the reciprocal-space resolution, as shown in the corresponding reflection.

Another notable observation of the ME-GID experiment was the low reciprocal-space resolution, particularly at high diffraction angles. This is attributed to the large footprint of the beam on the detector which leads to a smeared signal (Fig. 5 ▸). The effect is amplified by the short SDD, which is necessary when using moderate X-ray energies to cover a large volume of reciprocal space efficiently. The shorter the distance, the greater the relative uncertainty of the point of diffraction δ*d*/*d*_0_, where δ*d* is the cross section of the beam on the sample and *d*_0_ is the mean distance between the illuminated sample surface and the detector. This uncertainty has a direct effect on the accurate determination of the diffraction angle of a given feature and thus on the reciprocal-space resolution.

**Figure 6:**
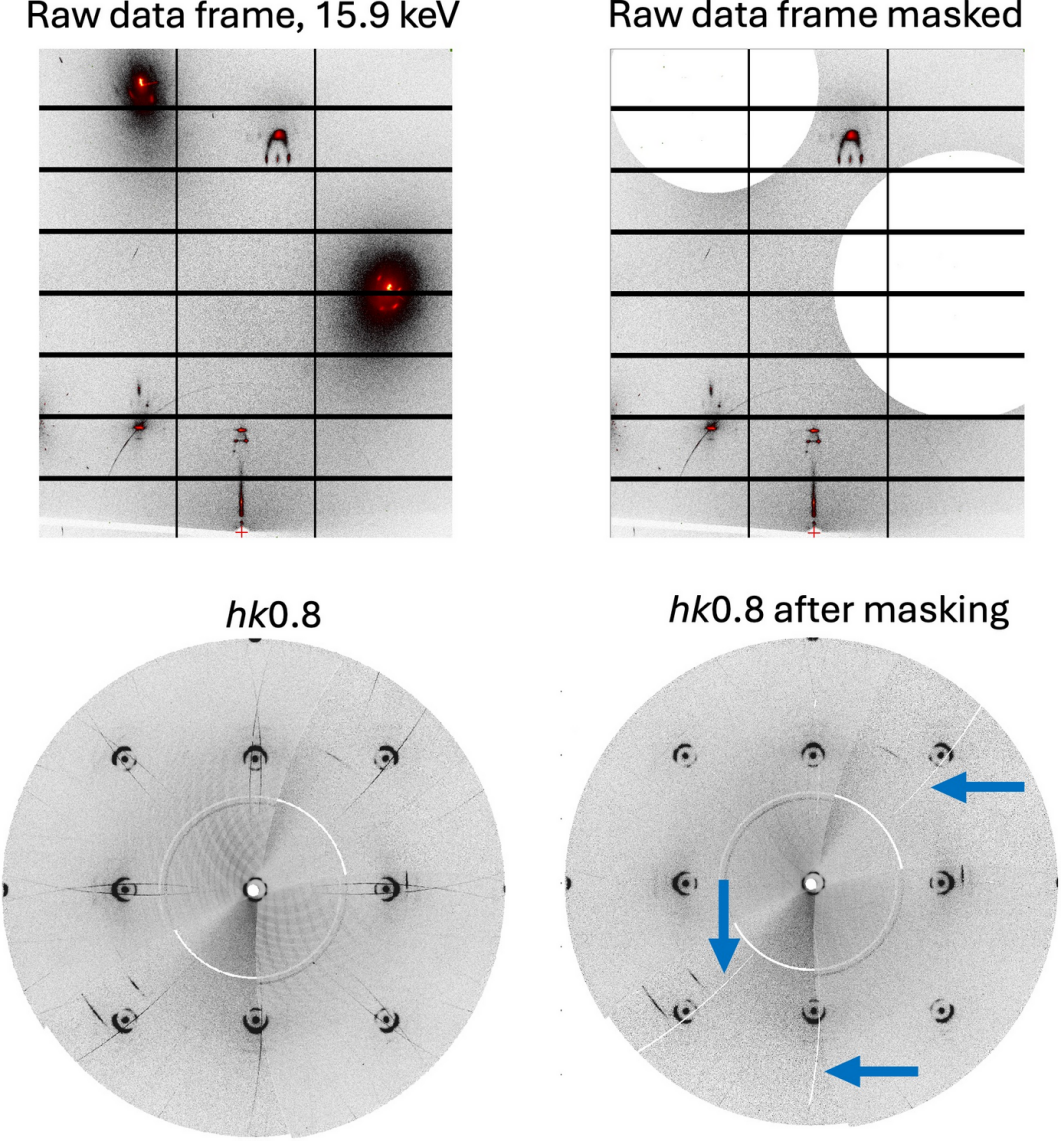
Raw scattering data frame collected at an SDD of 241 mm with a 15.9 keV X-ray beam on BM01 at ESRF, showing large halos around the strong Bragg reflections at the top left and the masked frame at the top right. The reconstructions of the *hk*0.8 layer before and after masking are shown at the bottom left and bottom right, respectively. The thin streaks penetrating the diffuse rings in the bottom left reconstruction are the results of the halos. The arrows on the bottom right reconstruction indicate the removal of the streaks as a result of masking. Note that the double streaks passing through the diffuse rings are a result of redundant measurements, which led to the filling of the masked regions.

Large halos with significant intensities were observed around strong substrate Bragg peaks in several data frames (Fig. 6 ▸). These halos only appear in frames where strong Bragg reflections hit the detector. In the reconstructions, the halos transform into thin features that follow the trace of the frames in which the strong Bragg peaks are recorded. The formation of these halos can be reasonably attributed to secondary air scattering (SAS) induced by strong Bragg peaks, as well as to fluorescence back-scattering originating from the fraction of strong peaks that, rather than being absorbed by the detector sensor, penetrate into the electronics behind it. A similar effect has been reported with other detector systems (Welberry *et al.*, 2005 ▸). To minimize the impact of the halos on the resulting reconstructions, the recorded data were masked with discs whose diameters were estimated by eye. As the halos occur only in frames containing intense substrate Bragg reflections, the missing information has a minimal impact on the results. Notably, the environment surrounding the reflection on a single frame varies depending on whether the reflection enters or leaves the Ewald sphere during the rotation. The symmetry-equivalent reciprocal-space regions are therefore affected differently, allowing us to fill in the missing information during the symmetry expansion. Narrow gaps can also be partially filled with data from symmetry expansion.

In addition to these halos, strong TDS from the substrate was observed in close proximity to the Bragg peaks due to the presence of strong substrate Bragg peaks, despite the relatively low temperature of 130 K. Given that TDS is a three-dimensional feature that overlaps with the features of interest, its complete elimination is challenging and therefore was not pursued.

Finally, the reciprocal-space coverage obtained in our ME-GID experiments was not sufficient for our chosen data evaluation method, three-dimensional difference pair distribution function (3D-ΔPDF) analysis. Figs. 4 ▸ and 7 ▸ illustrate the limitation of the experiment. Although single-crystal 3D-ΔPDF analyses have significantly lower requirements for reciprocal-space coverage than PDF investigations of polycrystalline materials (Weber & Simonov, 2012 ▸), the coverage obtained in the ME-GID experiments is inadequate for resolving small atomic shifts of the order of a few picometres, which are especially relevant for studying ferroelectric mater­ials.

In summary, the quantitative analysis of the diffuse scattering using ME-GID is hindered by several issues. These are:

(i) the shadowed regions at *l* < 0 and *l* ≃ 0,

(ii) the limited reciprocal-space resolution,

(iii) strong contributions from the substrate, and

(iv) the limited reciprocal-space coverage.

It is feasible to optimize ME-GID experiments in accordance with each of the aforementioned points, yet with limitations. For instance, reciprocal-space coverage could be improved by a shorter SDD, but at the expense of reciprocal-space resolution. The binning of reciprocal space collected at various detector positions could enhance the reciprocal-space coverage as well, though this approach may be limited by geometric constraints. Reciprocal-space resolution could be improved with a larger incident angle, which would reduce the footprint of the beam on the sample but would also reduce the surface sensitivity and increase the shadowed region around *l* ≃ 0. Conversely, a smaller incident angle would decrease the reciprocal-space resolution but could also reduce the cross-sectional area of the beam and the sample surface projected along the beam direction, resulting in a smaller usable fraction of the primary beam. The incident angle of 2° chosen for our ME-GID was therefore a compromise, enabling approximately a third of the available beam height of 0.5 mm (0.17 mm) to be utilized with a sample length of 5 mm.

Achieving high surface sensitivity in a GID experiment depends on the fraction of primary beam absorbed as it passes through the film and the beam’s penetration depth into the substrate. Note that surface sensitivity decreases as the incident angle increases. For an incident angle of 2° and an average absorption factor of the film for the corresponding energies, 3.9% of the primary beam is absorbed on its trajectory through the film at 12.8 keV, while 96% penetrates into the substrate; at 15.9 keV, the absorption within the film rises to 6.1%. The enhanced absorption at higher energy is owed to the fact that 15.9 keV is close to both the *K* edge of Sr and the *L* edge of Pb, whereas 12.8 keV is close to only the *L* edge of Pb. In this approximation, we disregard the absorption of the reflected beam, as the majority of the recorded signal exhibits a diffraction angle considerably larger than the incident angle, making the path of the diffracted beam within the film negligible.

Measurements taken just above the absorption edge significantly increase the surface sensitivity but also the background, due to fluorescence scattering as shown in Fig. 4 ▸. The primary beam and the diffracted beam in the substrate are attenuated by 90% after a beam path of only 150 µm at 12.8 keV and 290 µm at 15.9 keV. As a consequence, most of the diffracted intensity is attenuated, given the sample length of 5 mm. This is in agreement with our observation that the diffracted beam around *l* ≃ 0 is almost completely shadowed by the substrate.

### 3.3. HE-GID

The employment of high-energy X-rays offers a compelling solution to the limitations identified in the preceding section. In the context of grazing-incidence geometry, this approach provides several key advantages for the collection of three-dimensional diffuse scattering data from thin films:

(i) Extended reciprocal space and improved resolution. Small scattering angles enable the extension of reciprocal-space coverage and allow for long SDDs, which in turn enhance the reciprocal-space resolution by reducing the negative impact of the large footprint of the beam.

(ii) Access to lower *l* values. In the case of substrates with low absorption coefficients at high X-ray energies, it is feasible to facilitate scattering through the substrate, thus enabling access to reciprocal-space regions with small or even negative *l* values.

(iii) Reduction of the V-shaped blind region. The flatter curvature of the Ewald sphere at higher energies reduces the size of the V-shaped blind region typically observed along the rotation axis.

A potential drawback of high-energy X-rays is their increased penetration depth, which can reduce surface sensitivity. As Dippel *et al.* (2019 ▸) demonstrated, this effect can be effectively mitigated by employing ultra-small incident angles. In our experiments, we addressed the reduced beam cross section associated with such angles by focusing the beam to a 2 µm spot.

To identify the optimal incident angle for our experiments, we collected data at incident angles ranging from 0.01° to 0.1° in increments of 0.01°. As expected, higher incident angles led to increased substrate signals and decreased film signals due to greater X-ray transmission. Conversely, at the lowest angles, the reduced cross section of the beam on the sample resulted in a markedly weaker signal. From a comparative analysis of the diffuse scattering intensities, we determined 0.03° to be the optimal incident angle (Fig. 8 ▸). At this angle, the projected height of the sample (2.6 µm) slightly exceeds the beam height, ensuring complete interaction with the beam and full utilization of its intensity.

At 74 keV, the critical angles for total reflection are approximately 0.034° for STO and 0.041° for PTO (Seeck, 2014 ▸). Since the topmost layer of the superlattice is PTO, our chosen angle of 0.03° lies within the total external reflection regime. Nonetheless, scattering from the underlying layers, including the buffer and the substrate, was consistently observed. This can be attributed to imperfections in both the X-ray beam and the sample. The focused beam, with horizontal and vertical FWHMs of 30 and 2 µm, respectively, forms a short line focus whose precise alignment with the sample surface is limited. As a result, local variations in beam height, surface roughness and sample curvature can cause beam penetration into the sample bulk by locally violating the grazing condition or exceeding the critical angle. These effects are particularly pronounced at high X-ray energies due to the small critical angles and low attenuation, and have been previously discussed by Dippel *et al.* (2019 ▸).

Despite these challenges, the surface sensitivity achieved in the HE-GID experiments was approximately twice that of the ME-GID case. This is shown by the fact that the primary beam absorption within the film reached 8% at the incident angle of 0.03°. Moreover, 90% of the intensities were absorbed after approximately 5.5 mm of travel through the substrate, demonstrating that, even if a diffracted beam passes through the entire length of the 5 × 5 mm substrate, a significant observable intensity remains. However, since most diffraction signals pass through a fraction of the entire substrate length, a significantly lower absorption was observed.

The benefits of HE-GID experiments are clearly manifested in the reconstruction shown in Fig. 3 ▸. One key benefit was that the diffracted photons had enough energy to scatter through the substrate, allowing the collection of *l* ≤ 0 regions that could not be recorded with sufficient intensity in the ME-GID experiment. Furthermore, the reciprocal-space resolution was significantly enhanced compared with the ME-GID X-ray experiments, as discussed above. The large halos observed in the frames where the Bragg reflections from the substrate hit the detector were noted to be of similar intensity to those observed in the scattering data collected from the ME-GID experiment and were masked in the same manner.

**Figure 7:**
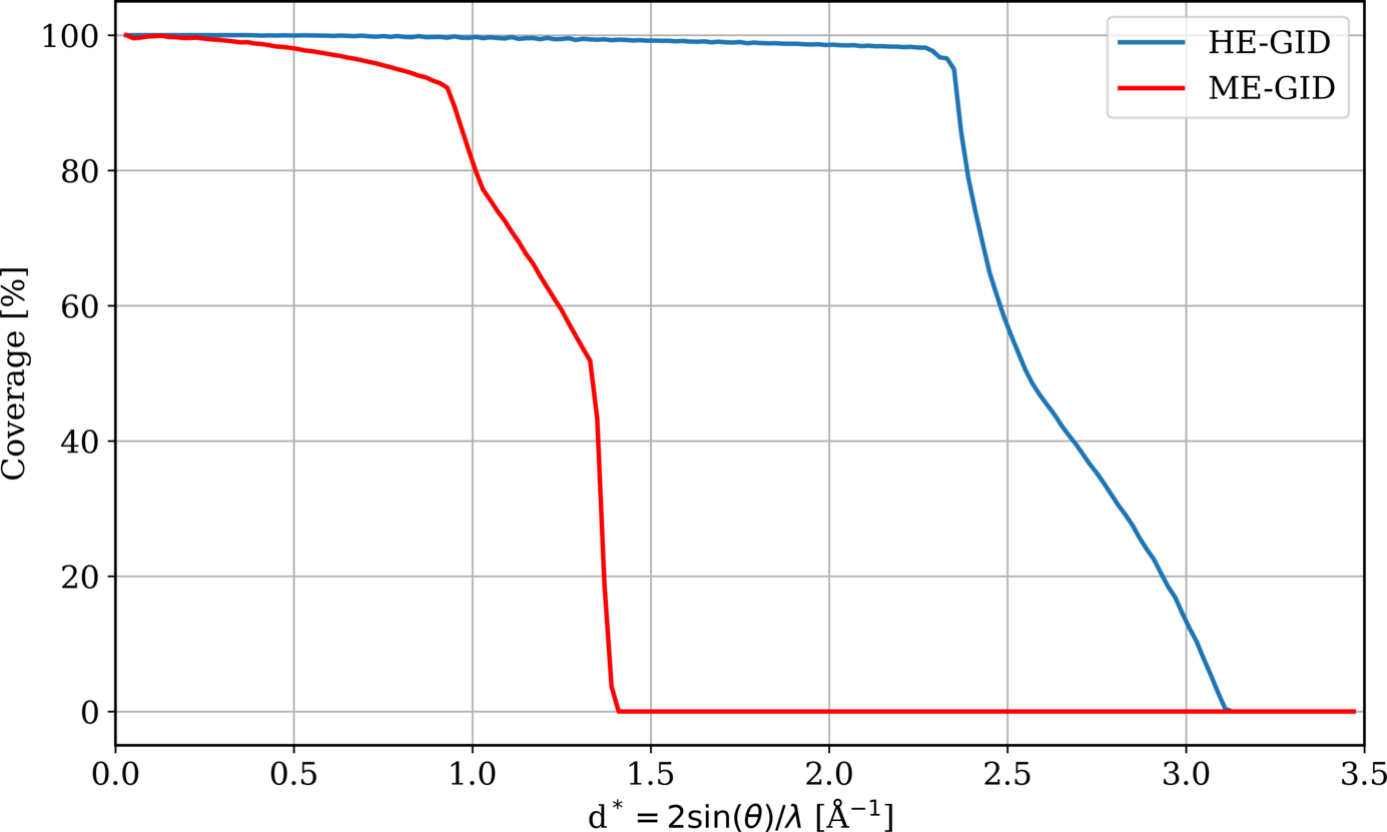
Reciprocal-space coverage of the ME-GID and HE-GID experiments depending on the scattering vector length 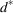 = 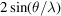, where θ is half of the diffraction angle and λ is the wavelength. For better comparability, the empty areas in the detector gaps of the ME-GID experiment were classified as observed and the V-shaped blind regions were kept unfilled in both cases. The influence of the blind regions on the data completeness at a given scattering vector length is evident in the ranges up to ∼0.9 Å^−1^ for ME-GID and ∼2.3 Å^−1^ for HE-GID. Note that the blind region has almost no effect on the HE-GID coverage, while it is very significant for the ME-GID case.

**Figure 8:**
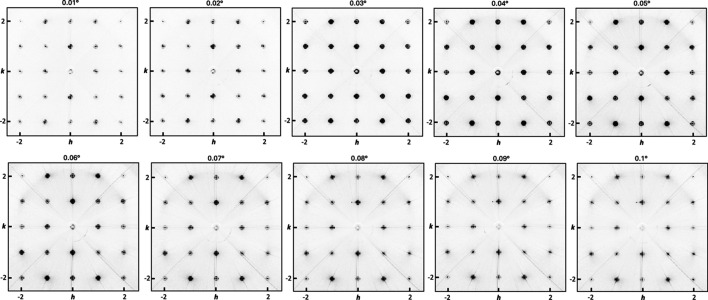
Reconstruction of *hk*0.96 layers, shown with a consistent grayscale, for a superlattice composed of seven-unit-cell-thick PTO and seven-unit-cell-thick STO layers, repeated 12 times, with a ten-unit-cell-thick SRO buffer layer on an STO substrate. Data were collected at an SDD of 493.7 mm using 74 keV X-rays at varying incident angles. The reconstruction at an incident angle of 0.03° exhibits the most pronounced first-order diffuse ring with sufficient intensity. At smaller incident angles, the scattering intensity decreases due to the reduced beam cross section on the sample, while at higher angles the surface sensitivity is compromised.

The high-energy radiation significantly expanded the reciprocal-space coverage compared with the ME-GID experiment, as illustrated in Fig. 7 ▸. Specifically, the V-shaped blind region in reciprocal space had a negligible impact on the completeness of the data compared with the ME-GID experiment due to the much lower curvature of the large Ewald sphere. The collection of ω scans to fill the gap was therefore less critical. In fact, the necessity for ultra-small incident angles in the HE-GID experiment rendered the employment of ω scans unsuitable for the completion of the V-shaped blind reciprocal-space region. The procedure of merging ϕ and ω scans, as described for moderate X-ray energies in the preceding section, would probably have been ineffective because the surface sensitivity would be significantly reduced and substrate scattering would become exceedingly strong. For example, at an incident angle of ω = 2° and an X-ray energy of 74 keV, the absorption of the primary beam in the film would be only 0.13%, as opposed to 8% at 0.03° and 0.026% at 10°, meaning that the surface sensitivity of the experiment would quickly approach 0 with increasing ω. Consequently, this approach was not attempted. In contrast, during the ME-GID experiments with 12.8 keV X-rays, the absorption of the primary beam in the film was 0.3% at the maximum incident angle of ω = 30°, a much higher absorption that maintained greater surface sensitivity than the HE-GID experiments.

**Figure 9:**
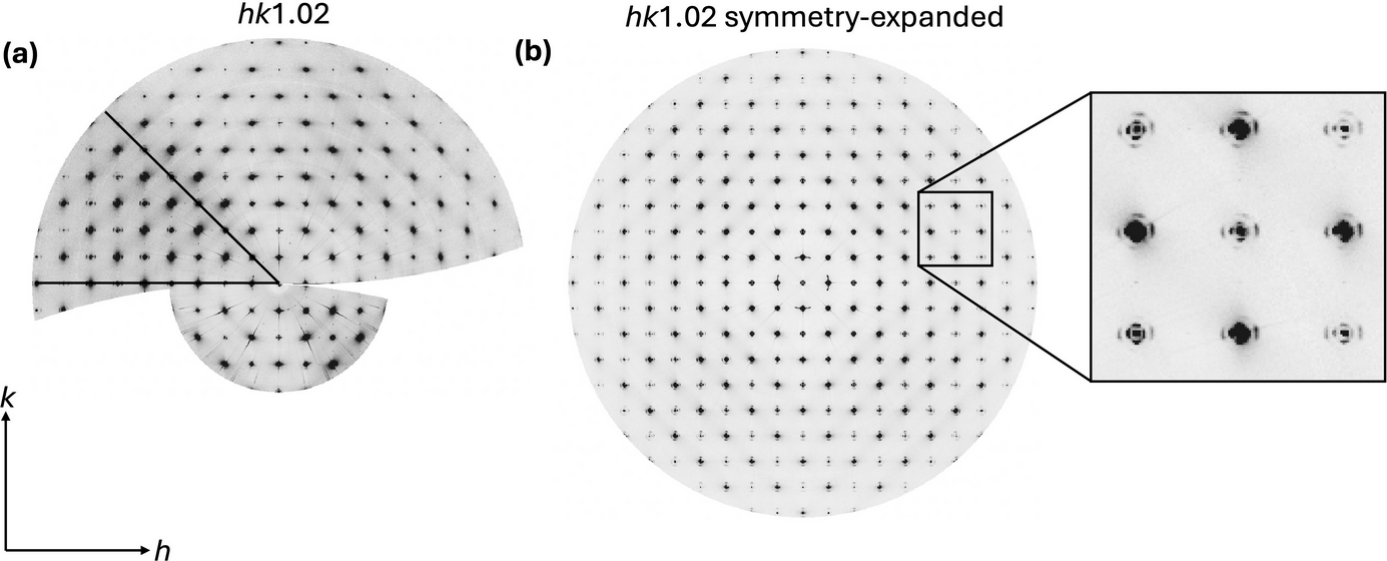
(*a*) Reconstructed *hk*1.02 layer collected from the HE-GID experiment at ultra-small-angle grazing-incidence geometry with an SDD of 491 mm. The modulation of intensity across the frames is evinced. The sector indicated with black lines represents the asymmetric unit of the Laue group 4/*mmm* with the strongest intensity. (*b*) Symmetry-expanded *hk*1.02 layer using the asymmetric unit indicated in panel (*a*) represented in a different contrast. The magnified inset on the right shows first-order and second-order diffuse rings around the CTRs. Note that regions outside the asymmetric unit were not used; instead, the full reciprocal space was reconstructed via symmetry expansion of the indicated subvolume in panel (*a*) as justified by the high crystal symmetry.

The advantages of HE-GID in tandem with ultra-small-angle grazing-incidence geometry are clear, but they can be even more pronounced with further experimental optimization. Specifically, collecting rotational step scans in ultra-small-angle grazing-incidence geometry requires high precision in aligning the sample normal parallel to the rotation axis of the instrument. Minor misalignment or mechanical instabilities that lead to a wobbling amplitude of the sample surface of the order of the incident beam angle, or sample shifts along the rotation axis of only a few hundred nanometres due to mechanical or temperature fluctuations, would result in significant variation of the cross section of the beam on the projected sample surface during a scan. Although the sample alignment procedure indicated an alignment accuracy of almost an order of magnitude better than the incident angle of the beam, significant modulation of intensity across the frames was still observed, as shown in Fig. 9 ▸(*a*). To address these issues, the asymmetric unit exhibiting the strongest substrate and diffuse features from the film was used for symmetry expansion, as depicted in Fig. 9 ▸(*b*). We assumed 4/*mmm* Laue symmetry for the film signal, given the *p*4*mm* layer symmetry of the substrate surface, and symmetry-expanded the asymmetric unit accordingly to fill the reciprocal space. Resolving the experimental instabilities would not only lead to more reliable data by minimizing intensity modulations but would also improve the efficiency of data processing, minimizing the need for extensive data corrections and symmetry assumptions.

### 3.4. Analysis of diffuse streaks using 3D-ΔPDF

In order to illustrate the applicability of our approach to the quantitative analysis of diffuse scattering from thin films, we report the results of a 3D-ΔPDF analysis of the diffuse streaks observed at half-integer *h*, *k* positions.

The 3D-ΔPDF is the auto-correlation function of local structure properties and it allows direct access to local order phenomena (Weber & Simonov, 2012 ▸). Such information is inaccessible from a conventional Bragg diffraction analysis, which is confined to the average structure. A 3D-ΔPDF analysis is conducted by Fourier transforming three-dimensional diffuse scattering data, which can be obtained by extracting the diffuse scattering from the total scattering data. In order to minimize Fourier transform truncation artifacts and achieve atomic resolution, it is essential to measure as complete a three-dimensional total scattering data set as possible. For this reason, our analysis was conducted exclusively with the HE-GID data, which offers superior reciprocal-space coverage over the ME-GID data. In the symmetry-expanded reciprocal space, separation of diffuse streaks was achieved by directly extracting the voxels containing them and integrating along the *h* and *k* directions. The background was removed by interpolating the surrounding background signal.

**Figure 10:**
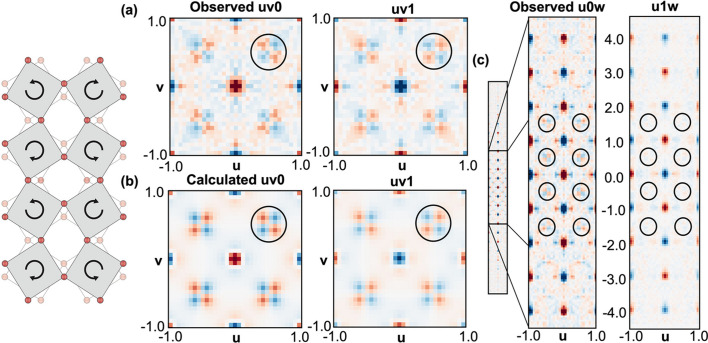
(*a*) Observed out-of-plane 3D-ΔPDF of the extracted half-integer streaks. (*b*) Refined 3D-ΔPDF of the *a*^0^*a*^0^*c*^−^ tilt pattern of oxygen octahedra viewed along the out-of-plane direction illustrated in the model on the left. (*c*) Observed 3D-ΔPDF of the extracted half-integer streaks in the *u*0*w* and *u*1*w* layers. Note that the encircled features are not covered by the *a*^0^*a*^0^*c*^−^ model. In the 3D-ΔPDF map, red colors denote positive and blue colors negative 3D-ΔPDF values. Positive 3D-ΔPDF values mean that the probability of finding scattering densities separated by the corresponding vector is higher than in the average structure (= positive correlation), whereas negative values imply lower probability (= negative correlation).

The extracted half-integer streaks were Fourier transformed to create 3D-ΔPDF maps (Fig. 10 ▸). In the following, the *u* and *v* autocorrelation space coordinates correspond to the unit-cell size along the common **a** and **b** directions, while *w* coordinates are expressed in terms of the average unit-cell size of the film along the out-of-plane direction. In the *uv*0 layer of the resulting 3D-ΔPDF map, among other observations, a prominent four-set checkerboard pattern was observed around (0.5, 0.5, 0) and at symmetry-equivalent positions. All of the observed features are also present in the *uv*1 layer, albeit with an inverted sign [Fig. 10 ▸(*a*)]. In general, the patterns exhibit a consistent behavior across all layers with *w* = even and inverted correlations for *w* = odd. There is a general trend that the intensity of the features decreases with increasing *w*, indicating a slowly decreasing correlation of the underlying structural features along the out-of-plane direction. The patterns within the *uv* planes could be associated with a gear-like anti-phase tilt pattern of the oxygen octahedra, commonly observed in perovskite structures. The sequence of inverted correlations along the 00*w* direction indicates that neighboring planes along the out-of-plane direction show the same tilt pattern but with an opposite sense of rotation [Fig. 10 ▸(*c*)]. The four-set checkerboard pattern is also observed around (0.5, 0, 0.5) in the *u*0*w* layer [Fig. 10 ▸(*c*)], which signifies the presence of octahedral tilts in the in-plane axes. Conversely, the marked absence or substantial reduction in intensity of the pattern in the *u*1*w* layer implies the presence of both out-of-phase and in-phase tilts in nearly equal proportions, thereby resulting in a net zero ΔPDF intensity.

As a proof of principle for quantitative analysis of the local structure, we restrict ourselves here to modeling the 3D-ΔPDF features near *u* = 0.5, *v* = 0.5 and symmetry equivalents, *i.e.* the anti-phase tilt correlations along the out-of-plane direction. This simplification corresponds to an *a*^0^*a*^0^*c*^−^ tilt model in Glazer notation (Glazer, 1972 ▸; Glazer, 1975 ▸). The map was calculated using *Yell*, a program for the refinement of disorder models against single-crystal diffuse scattering data (Simonov *et al.*, 2014 ▸). Agreement of the refinement with the observed 3D-ΔPDF is evident in Fig. 10 ▸(*b*). The refinement suggests that the amplitude of the tilt is 6.7°.

## 4. Conclusion

We explore the feasibility of investigating the local structure of thin films by measuring large reciprocal-space volumes with the grazing-incidence diffraction method and extracting local structural order information. The proposed method is non-invasive and non-destructive and provides quantitative local order information at the atomic level that is representative of the entire sample. Consequently, it is a valuable complement to the established imaging methods, which probe only a very small volume of the material under investigation, and to scattering methods that average the sample structure but are limited in spatial resolution.

The application of 74 keV high-energy X-rays demonstrated remarkable advantages over conventional diffraction experiments conducted with moderate energies. The shorter wavelength enabled significantly broader reciprocal-space coverage, allowing the extraction of real-space properties with atomic resolution down to a few picometres. By employing smaller scattering angles, focused beams and extended SDDs, the reciprocal-space resolution was markedly enhanced, minimizing artifacts such as smeared signals associated with large beam footprints on the detector in grazing-incidence diffraction measurements with short SDDs. Notably, the low absorption of the substrate in the HE-GID experiment enabled *hk*0 layer observations, which provided critical information on in-plane components of atomic displacements (Fig. 3 ▸) that are independent of out-of-plane components. The significance of the data extracted from the *hk*0 layer for the identification of the structural properties of domain walls has been elucidated by Zatterin *et al.* (2024 ▸) with remarkable clarity. However, the X-ray energy employed in their experiments results in substantial attenuation of the region around the *hk*0 layer due to substrate absorption, which has the effect of reducing the visibility of weak and moderate scattering signals. In such instances, high-energy experiments represent a valuable approach for the acquisition of more detailed information. Moreover, focusing the high-energy beam down to a few micrometres allowed a very small incident angle to be applied without significant loss of primary intensity. In the ultra-small-angle grazing-incidence geometry, the surface sensitivity of the experiment, an inherent problem of high X-ray energy experiments, could as a consequence be maintained at a comparable level to that observed during experiments with moderate energies.

This work not only provides a novel experimental strategy for collecting three-dimensional diffuse scattering data but also paves the way for advances in experimental design to collect such data with even greater resolution and quality. For instance, increasing the thickness or bilayer stack sizes of the superlattices can enhance the film signal relative to the substrate, making it easier to extract signals close to substrate reflections, such as diffuse rings around the CTRs. Adjusting the X-ray energy to balance surface sensitivity, reciprocal-space resolution and coverage also holds promise. By tuning energies above and below the absorption edges, the balance between surface sensitivity, reciprocal-space resolution and coverage can be finely controlled, enabling the detection of even the weakest signals. Mechanical stability and sample alignment during HE-GID experiments remain areas for improvement, as addressing intensity modulation due to misalignment or instabilities could enhance data reliability. Larger sample sizes or incident angles may be employed as well to compensate for such instabilities. The collection of data with reduced intensity modulations across the frames will allow for the investigation of systems with low symmetry, which may be limited in symmetry expansion during data processing.

In conclusion, this pioneering study demonstrates that HE-GID is a powerful tool for extracting quantitative local structural information from single-crystalline thin films with atomic resolution. Its combination of surface sensitivity, high reciprocal-space resolution, high coverage and experimental flexibility makes it a versatile approach for probing the local structure of epitaxial thin films. The adaptability and compatibility of the method with the currently existing tools for processing and analyzing data make it robust. With continued refinement and optimization, HE-GID is poised to make significant contributions to the study of local structures in complex epitaxial thin films.

## Figures and Tables

**Table 1:** Comparison of experimental parameters in ME-GID and HE-GID experiments

	ME-GID	HE-GID
Energy	12.8 keV, 15.9 keV	74 keV
Incident angle	2°	0.03°
SDD	143 mm, 241 mm	493.7 mm, 850 mm
Size of the beam	0.5 × 0.5 mm	30 µm (horizontal) × 2 µm (vertical)
Beam divergence	0.2°	0.003°
Transmission length in the film	150 µm (12.8 keV), 290 µm (15.9 keV)	5.5 mm
Minimum unshaded *l*	0.17	0.012
